# Effect of perioperative hypotension on long-term orthopaedic surgery recovery: A review

**DOI:** 10.6026/973206300210768

**Published:** 2025-04-30

**Authors:** Kumari Sneha, Srirupa Mandal, Kalyan Kumar Saha, Samrat Smrutiranjan Sahoo, Vinod Kumar, Mukesh Kumar, Sommya Kumar

**Affiliations:** 1 Department of Anaesthesia, AIIMS, Kalyani, West Bengal, India; 2 Department of Cardiology, AIIMS, Kalyani, West Bengal, India; 3 Department of Orthopedics, AIIMS, Kalyani, West Bengal, India; 4 Department of Urology, AIIMS, Kalyani, West Bengal, India; 5 Department of Dentistry, AIIMS, Kalyani, West Bengal, India; 6 Department of Dentistry, AIIMS Deoghar, Jharkhand, India

**Keywords:** Hypotension, orthopedic procedures, postoperative care, patient safety, recovery of function

## Abstract

The occurrence of perioperative hypotension is common among orthopedic surgical patients and it results in negative long-term outcomes. Therefore, it is of interest to review the effect of perioperative hypotension on long-term orthopaedic surgery recovery. The condition showed increased danger among patients experiencing delirium along with cardiovascular problems. The main factors that resulted in perioperative hypotension consisted of hypertension alongside heart diseases combined with blood loss and the use of beta-blockers and extended surgical durations. Early detection along with proper management requires immediate attention because of their crucial importance. The optimization of blood pressure during surgical periods leads to better patient security together with enhanced functional outcomes. The execution of orthopedic procedures needs to focus on maintaining stable blood pressure levels according to clinical guidelines.

## Background:

Blood pressure is a complex and multifaceted physiological parameter with far-reaching implications for the human body. As such, precise blood pressure measurement and prompt management of any fluctuations are crucial, particularly during the perioperative period. However, in the older population, pre-existing comorbidities combined with the stress of surgery and anesthesia can lead to significant variations in perioperative blood pressure, making management increasingly challenging. Furthermore, the reduced physiological reserve of elderly patients under surgical stress limits their ability to adapt to blood pressure fluctuations, thereby complicating management [1]. Perioperative blood pressure management lacks standardized targets, largely due to the complex and multifaceted physiology of blood pressure regulation [2]. Despite the importance of blood pressure (BP) monitoring in perioperative care, there is currently no consensus on optimal BP targets for individual patients under anesthesia and surgery. In contrast, primary care has established clear BP targets for hypertension (130/80 mmHg) [3]. It is notable that primary care, where BP is checked less frequently, has defined therapeutic targets for a large patient population, raising the question of whether similar targets can be established for perioperative care. The body utilizes various short-term and long-term mechanisms to control blood pressure, with each organ employing auto-regulatory mechanisms to maintain its own blood flow [4]. Perioperative hypotension during with after non-cardiac surgery is a complex issue with multiple contributing factors, including patient characteristics, pharmacological interventions and procedural elements [5]. Despite frequent or continuous hemodynamic monitoring, intraoperative hypotension can still occur. Moreover, postoperative hypotension is often severe, prolonged, and may go undetected with conventional intermittent vital sign monitoring [6]. As a result, preventing perioperative hypotension poses a significant physiological challenge for anesthesiologists [7]. The primary objective of perioperative blood pressure management is to ensure adequate organ perfusion while minimizing the risk of organ damage. Uncontrolled blood pressure fluctuations can lead to ischemia and other adverse post-surgical events [8]. Therefore, it is of interest to explore the relationship between blood pressure (BP) and outcomes, as well as the effects of BP management strategies on perioperative outcomes.

## Materials and Methods:

The inclusion criteria were framed as per internationally standardized PICOS framework, as recommended by PRISMA guidelines:

## Participants/population:

The study population included patients who underwent orthopaedic surgery.

## Intervention:

Impact of perioperative hypotension on long-term orthopaedic surgery outcomes and effect on patient safety and functional recovery.

## Comparator(s)/control:

Studies of any of the above-mentioned interventions were included, including studies with no comparator group.

## Outcome:

Primary outcomes of the study were to determine the association between perioperative hypotension among patients who underwent orthopaedic surgery and long-term orthopaedic surgery outcomes w.r.t patient safety and functional recovery.

## Study design:

The review included all types of experimental studies, observational studies and case series which have reported the outcomes of the above-mentioned treatment therapy.

## Inclusion criteria:

Studies conducted anywhere in the world and articles published after 2010 through December 2024 was included in the study. Only those studies published in English language, academic peer-reviewed journals were included in the review.

## Exclusion criteria:

Case studies was excluded from the study. Studies conducted on animals were excluded from the study.

## Literature search:

A systematic literature search was performed in PubMed, Embase, clinical trial.gov, google scholar and Cochrane Library through December 2024 in the English language by two independent authors using a structured search strategy. The searches were screened by the references of selected articles to find those that did not appear in the search databases. Additional references were not obtained by free internet search from Google as the number of studies was large. The detail search strategy is given in Table 1.

## Process of screening and selection of articles:

All the citations along with the title and abstract were added to a specified endnote library and final list of studies to be screened for inclusion in the study was prepared by removing the duplicates. Two researchers carefully screened the articles by assessment of the title and thorough reading the abstracts to shortlist the studies which are likely to satisfy the inclusion criteria of the review. Attempts were made to obtain full-text articles for all these shortlisted studies, and thorough assessment was done for the satisfaction of inclusion and exclusion criteria. Studies not satisfying inclusion criteria were excluded further. The list of excluded studies and the reasons for exclusion were presented in the "characteristics of excluded studies" table. "PRISMA flow chart" was used to clearly represent the screening and selection process (Figure 1 - see PDF).

## Data extraction:

Data was thoroughly read through and were extracted from included studies was extracted manually on to a structured data extraction form. The number of patients *i.e.*, incidence of patients with atrial fibrillation among diabetic patients, any reported gender prevalence as well as any associated factors that increase the risk of atrial fibrillation among diabetic individuals were reported.

## Risk of bias in individual studies:

The methodological quality of studies included in the systemic review was assessed according to Fowkes and Fulton quality assessment [8].

## Study outcome:

A total of 18,822 participants were included in the 8 studies that were conducted from 2010 to 2024 (Table 1), which examined various orthopedic surgical procedures, with a total of 7,191 participants undergoing procedures such as traumatic hip fracture surgery (n = 689), orthopedic upper extremity surgery with brachial plexus blockade (n = 2,152), proximal femoral fracture repair (n = 1,131), and neck-of-femur (NOF) fractures (n = 276). Additionally, 605 participants underwent orthopaedic and thoracic surgery.

One large cohort study included 11,085 participants that underwent hip surgery. These studies were conducted in various countries, including Greater Britain, Korea, China, India, and the United Kingdom. Table 2 presents the evaluation of studies reporting the impact of perioperative hypotension on long-term orthopaedic surgery outcomes. The studies, conducted between 2011 and 2024, examined the incidence of perioperative hypotension, risk factors, and outcomes. The incidence of perioperative hypotension ranged from 31.1% to 68%, with various definitions of hypotension used across studies. Risk factors for perioperative hypotension included hypertension, heart disease, perioperative blood loss and concurrent use of beta blockers, longer duration of surgery, lower preoperative systolic blood pressure, and higher preoperative heart rate. The outcomes associated with perioperative hypotension included postoperative delirium, postoperative complications, mortality, and prolonged length of stay. The studies suggest that perioperative hypotension is a significant predictor of adverse outcomes in orthopaedic surgery patients with brief description of studies as follows Paul *et al.* (2024) [8] studied 276 orthopedic surgery patients and found a 68% incidence of perioperative hypotension, which was associated with postoperative delirium, morbidity, mortality, and prolonged hospital stays. Baek *et al.* (2023) [9] examined 2,152 patients undergoing orthopedic upper extremity surgery and found a higher risk of perioperative hypotension with dexmedetomidine use. Duan *et al.* (2023) [10] investigated 605 patients and found a significant association between hypotension duration and postoperative delirium. Mishra *et al.* (2023) [11] studied 122 patients and found a 54.9% incidence of intraoperative hypotension, which was significantly associated with postoperative complications. White *et al.* (2016) [12] analyzed 11,085 patients and found associations between lower blood pressure during surgery and increased mortality risk. Wang *et al.* (2015) [13] investigated 103 patients and found a J-shaped association between mean arterial pressure and postoperative delirium. Kim *et al.* (2015) [14] examined patients undergoing hip fracture surgery and found an association between intraoperative hypotension and postoperative cardiovascular complications. Wood *et al.* (2011) [15] investigated patients undergoing hip fracture surgery and found correlations between perioperative hypotension, anesthesia type, and bupivacaine volume.

## Discussion:

Intraoperative hypotension is a significant concern in non-cardiac surgery under general anesthesia, as it has been linked to various adverse outcomes, including postoperative mortality, [16, 17] myocardial injury after non-cardiac surgery (MINS),[18] myocardial infarction, [19] cardiogenic shock, [20] acute renal failure, [21] delirium, [22] and stroke [23]. This is particularly relevant for patients undergoing non-cardiac surgery, where the risk of these complications is heightened. The body has mechanisms to protect vital organs, such as the brain, heart, and kidneys, from hypotension-induced hypoperfusion through blood flow auto-regulation. However, other organ systems, including splanchnic organs like the stomach, liver, and pancreas, are more vulnerable due to their limited autoregulation capacity. As a result, close monitoring of blood pressure using invasive or non-invasive methods is crucial in perioperative and critical care medicine to ensure patient safety and maintain perfusion pressure. Early recognition and treatment of imminent hypotension are increasingly important in reducing the severity of hypotension [24].

The present analysis found that outcomes associated with perioperative hypotension included postoperative delirium, postoperative complications, mortality, and prolonged length of stay. The studies suggest that perioperative hypotension is a significant predictor of adverse outcomes in orthopaedic surgery patients. Analysis reported that Paul *et al.* (2024) [8] investigated the impact of perioperative hypotension on outcomes in 276 orthopedic surgery patients, finding a 68% incidence of hypotension and associations with postoperative delirium, morbidity, mortality, and prolonged hospital stays. Baek *et al.* (2023) [9] examined the risk of perioperative hypotension in 2,152 patients undergoing orthopedic upper extremity surgery, finding a higher risk with dexmedetomidine use. Duan *et al.* (2023) [10] investigated the relationship between hypotension duration and postoperative delirium in 605 patients, finding a significant association. Mishra *et al.* (2023) [11] studied the impact of intraoperative hypotension on postoperative complications in 122 patients, finding a 54.9% incidence of hypotension and a significant association with complications. White *et al.* (2016) [12] analyzed mortality rates in 11,085 patients, finding associations between lower blood pressure during surgery and increased mortality risk. Wang *et al.* (2015) [13] investigated the relationship between mean arterial pressure and postoperative delirium in 103 patients, finding a J-shaped association. Kim *et al.* (2015) examined the impact of intraoperative hypotension on postoperative cardiovascular complications in patients undergoing hip fracture surgery. Wood *et al.* (2011) investigated the incidence and predictors of perioperative hypotension in patients undergoing hip fracture surgery, finding correlations with anesthesia type and bupivacaine volume. Intra-operative hypotension in elderly patients with chronic organ pathology increases their susceptibility to postoperative cardiac dysfunction and mortality. A study by Paul *et al.* reported that POH is associated with increased postoperative complications. Hypertension, heart disease, and perioperative blood loss are significant risk factors for POH. POH is significantly associated with adverse outcomes, including delirium; prolonged hospital stays, and elevated 30-day morbidity. The occurrence of POH, rather than its duration, is a significant factor in developing complications. Another study by Kim *et al.* also found that postoperative cardiovascular complications were related to frequent intraoperative hypotension (P <0.05) [14]. To counteract intra-operative hypotension, clinicians often employ strategies such as administering vasopressor agents and intravenous fluids. However, these strategies have potential side effects. Vasopressor agents can increase myocardial work and oxygen demand by rapidly increasing venous return (alpha-agonists) or positive ino-chronotropy (beta-agonists). Intravenous fluids, on the other hand, can render elderly patients susceptible to peripheral and pulmonary edema, particularly after discontinuation of anesthesia when systemic vascular tone is restored. Avoiding intra-operative hypotension can reduce the need for vasopressor or intravenous fluid administration, as suggested by our analysis [25]. Despite its clinical significance, there is no universally accepted definition of intraoperative or postoperative hypotension. Hypotension is typically defined using absolute or relative thresholds for various blood pressure components, often specifying duration of exposure. A systematic review by Bijker *et al.* identified 140 different definitions of intraoperative hypotension across 130 articles, highlighting the variability in definitions. The most common definition was a 20% reduction in systolic blood pressure from baseline. Applying these definitions to a large retrospective cohort of over 15,000 adults undergoing noncardiac surgery revealed substantial variations in the incidence of intraoperative hypotension depending on the definition used. For example, using a 20% reduction in systolic blood pressure, the incidence of intraoperative hypotension was 93% for a 1-minute exposure, 88% for a 5-minute exposure, and 78% for a 10-minute exposure. In contrast, applying an absolute mean arterial pressure threshold of 65 mmHg yielded an incidence of 65% for a 1-minute exposure, 49% for a 5-minute exposure, and 31% for a 10-minute exposure [23]. A systematic review by Spahn *et al.* [261] investigated perioperative anemia in patients undergoing major orthopedic surgery. Preoperative anemia was prevalent (24-44%), and postoperative anemia was even more common (51-87%). Perioperative anemia was associated with increased blood transfusions, postoperative infections, poorer recovery, and higher mortality. Treatment of preoperative anemia with iron and erythropoietin, and perioperative cell salvage, reduced the need for blood transfusions and may improve patient outcomes. A study carried out by Maheshwari *et al.* [22] among 1,083 postoperative patients admitted to the surgical care unit found that 35% developed delirium within the first 5 days. Intraoperative hypotension, defined as a mean arterial pressure <65 mmHg, was moderately associated with higher odds of postoperative delirium. Specifically, a 1 mmHg increase in intraoperative hypotension was linked to an 11% increased hazard of delirium. Additionally, postoperative hypotension was significantly associated with delirium, with a 10mm Hg reduction in mean arterial pressure increasing the hazard of delirium by 12% and the study concluded that both intraoperative and postoperative hypotension are associated with delirium in critical care patients.

## Conclusion:

Hypotensive conditions during surgery enhance the potential for negative results such as delirium along with medical complications which can be fatal. The major risk factors which contribute to perioperative hypotension are hypertension combined with cardiac diseases as well as extended operative periods. Dedicated blood pressure management before operations becomes crucial to provide better safety and recovery results for patients.

## Figures and Tables

**Table 1:** Details studies reporting impact of perioperative hypotension on long-term orthopaedic surgery

**Author**	**Year**	**Type of study**	**Country**	**No. of participants in study group**	**Surgery**
Paul *et al.* [8]	2024	Case-control and cohort study	Greater Britain	276	Neck-of-femur (NOF) fractures
Baek *et al.* [9]	2023	Single-center retrospective study	Korea	2152	Orthopedic upper extremity surgery with brachial plexus blockade
Duan *et al.* [10]	2023	Retrospective observational cohort study	China	605	Orthopaedic and Thoracic surgery
Mishra *et al.* [11]	2023	Retrospective study	India	122	Traumatic hip fracture surgery
Wang *et al.* [13]	2015	Secondary analysis of a RCT study		103	elderly hip fracture patients
Kim *et al.* [14]	2015	Retrospective study	Korea	464	Elderly patients (aged 65 years or older) undergoing hip fracture surgery
White*et al.* [12]	2016	Retrospective analysis	United Kingdom	11085	Hip fracture
Wood *et al.* [15]	2011	Retrospective, observational study	United Kingdom	1131	Proximal femoral fracture repair

**Table 2:** Evaluation of studies reporting impact of perioperative hypotension on long-term orthopaedic surgery

**Author**	**Incidence of perioperative hypotension (POH)/Association of hypotension with outcome**	**Risk factors for POH**	**Outcome**
Paul *et al.* (2024) [8]	68% (188/276); Preoperative hypotension: 9.78% Intraoperative hypotension: 48.55% Postoperative hypotension: 24.63% (MAP < 65 mmHg) and 34.42% (systolic BP < 80%)	Hypertension (OR: 1.330) Heart disease (OR: 2.768) perioperative blood loss (OR: 1.42)	Postoperative delirium (RR: 2.037), Postoperative 30-day morbidity (RR: 4.008), Postoperative 30-day mortality (RR: 6.12), 365-day mortality (RR: 2.224), Postoperative delay in mobilisation (RR: 1.329), Prolonged length of stay (RR: 1.273)
Baek *et al.* (2023) [9]	1. Incidence of perioperative hypotension: Defined as systolic blood pressure (SBP) < 90 mmHg or mean blood pressure (MBP) < 60 mmHg 2. Odds ratio for hypotension: 5.68 (95% CI, 2.86 to 11.28) for dexmedetomidine group (group D) compared to non-sedated group (group N)	Concurrent use of beta blockers, Longer duration of surgery, Lower preoperative systolic blood pressure (SBP), Higher preoperative heart rate	Increased risk of hypotension: Associated with dexmedetomidine use in patients undergoing orthopedic upper extremity surgery with brachial plexus blockade.
Duan *et al.* (2023) [10]	NM	Long Duration of Hypotension: ≥ 5 minutes of MAP ≤ 65 mmHg (adjusted OR 3.93; 95% CI: 2.07-7.45; P < 0.001)	Incidence of Postoperative Delirium (POD): 14.7% (89 cases out of 605) within three days after surgery, Duration of Hypotension: ≥ 5 minutes of mean arterial pressure (MAP) ≤ 65 mmHg was associated with increased POD incidence, Increased Incidence of POD: associated with long duration of hypotension (MAP ≤ 65 mmHg for ≥ 5 minutes)
Mishra *et al.* (2023) [11]	Incidence of intraoperative hypotension: 54.9% (67 patients)	Associated with both intraoperative hypotension and postoperative complications	Incidence of postoperative complications: 56.7% (38 patients) in those with intraoperative hypotension, 34.5% in those without hypotension (P < 0.01). Correlation between intraoperative hypotension and postoperative complications: Patients with intraoperative hypotension experienced postoperative complications more frequently than those with stable vital signs (56.7% vs. 34.5%, P < 0.01).
White *et al.* (2016) [12]	Not mentioned	Lower systolic blood pressure (SBP) and mean arterial pressure (MAP) during surgery; Higher volume of subarachnoid bupivacaine administered	Mortality Rates: 1.5% (165/11,085) within 5 days after surgery, 5.1% (563/11,085) within 30 days after surgery Association between Blood Pressure and Mortality: - Lower SBP and MAP during surgery associated with increased risk of mortality, Odds ratio (95% CI) for mortality within 5 days: 0.983 (0.973-0.994) for each 5 mmHg increment in SBP, Odds ratio (95% CI) for 30-day mortality: 0.968 (0.951-0.985) for each 5 mmHg increment in SBP. Correlation between Bupivacaine Volume and Blood Pressure Weak correlation between lowest SBP after intrathecal local anaesthetic and volume of subarachnoid bupivacaine, Mean 20% relative fall in SBP correlated with an administered volume of 1.44 ml hyperbaric bupivacaine
Wang *et al.* (2015) [13]	J-shaped association between absolute levels of mean surgery MAP (msMAP) and PD risk	Not mentioned	Incidence of PD: 22% (23 patients) developed PD on day 2; role of mean arterial blood pressure (MAP) on postoperative delirium (PD): Very high msMAP levels: ≥80 mmHg, higher msMAP imparted greater PD risk (OR = 2.28 per 10 mmHg msMAP increase; 95% CI: 1.11-4.70), Very low msMAP levels: <80 mmHg, higher msMAP was associated with lower PD risk (OR = 0.19 per 10 mmHg increase; CI: 0.05-0.76)
Kim *et al.* (2015) [14]	Frequent intraoperative hypotension (P < 0.05): Postoperative cardiovascular complications	Not mentioned	Cardiovascular complications: 4.7%
Wood *et al.* (2011) [15]	Absolute hypotension (lowest systolic blood pressure < 90 mmHg): 31.1% (general anesthesia), 11.3% (spinal anesthesia with ≤ 1.5 ml bupivacaine), Relative hypotension (> 20% fall in systolic blood pressure from baseline): 83.9% (general anesthesia), 26.8% (spinal anesthesia with ≤ 1.5 ml bupivacaine)	Not mentioned	Volume of subarachnoid hyperbaric bupivacaine: Correlated with fall in systolic blood pressure (p = 0.004); Mean peri-operative fall in haemoglobin concentration
